# Inferring robust gene networks from expression data by a sensitivity-based incremental evolution method

**DOI:** 10.1186/1471-2105-13-S7-S8

**Published:** 2012-05-08

**Authors:** Yu-Ting Hsiao, Wei-Po Lee

**Affiliations:** 1 Department of Information Management, National Sun Yat-sen University, 70, Lienhai Road, Kaohsiung, Taiwan

## Abstract

**Background:**

Reconstructing gene regulatory networks (GRNs) from expression data is one of the most important challenges in systems biology research. Many computational models and methods have been proposed to automate the process of network reconstruction. Inferring robust networks with desired behaviours remains challenging, however. This problem is related to network dynamics but has yet to be investigated using network modeling.

**Results:**

We propose an incremental evolution approach for inferring GRNs that takes network robustness into consideration and can deal with a large number of network parameters. Our approach includes a sensitivity analysis procedure to iteratively select the most influential network parameters, and it uses a swarm intelligence procedure to perform parameter optimization. We have conducted a series of experiments to evaluate the external behaviors and internal robustness of the networks inferred by the proposed approach. The results and analyses have verified the effectiveness of our approach.

**Conclusions:**

Sensitivity analysis is crucial to identifying the most sensitive parameters that govern the network dynamics. It can further be used to derive constraints for network parameters in the network reconstruction process. The experimental results show that the proposed approach can successfully infer robust GRNs with desired system behaviors.

## Background

Gene regulatory networks (GRNs) are essential for controlling cellular metabolism and the organismal development. Under the command of transcription factors (TFs), each gene influences the activity of the cell by generating messenger RNA (mRNA) that guides the synthesis of proteins by ribosomes in the cytoplasm (the location in the cell where biochemical reactions and molecular events take place). Gene network modeling uses gene expression data to characterize the phenotypic behavior of a system under study. With the reconstructed networks, biologists can generate and test hypotheses to further understand the complex phenomena that occur in nature systems and to explore the dynamics of those systems.

**Figure 1:**
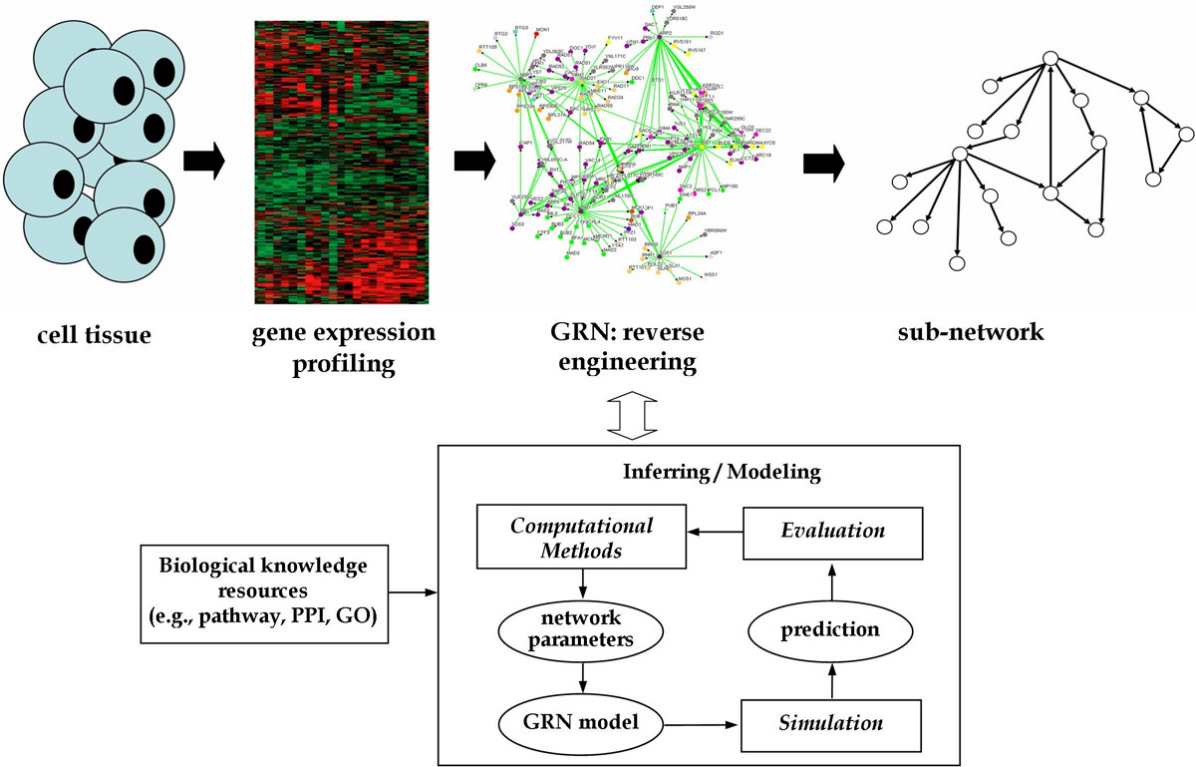
**Reverse-engineering a GRN**. General procedure for reverse engineering a gene regulatory network.

Modeling GRNs manually on the basis of the experimentally measured time-series data takes a considerable amount of time. Therefore, an automated reverse-engineering procedure is recommended [1,2]. This procedure involves altering the gene network in some way, observing the outcome, and using computational methods to infer the underlying principles of the network. To derive a realistic model, available domain knowledge (including functional and structural information) can be integrated into the computational methods. Figure 1 illustrates the general procedure of inferring GRNs from quantitative expression data. The inferring/modeling block of this figure indicates the computational procedure used to derive network parameters for a given model, to build and simulate the model, and to evaluate it by comparing the behavior of the inferred model with the original data set. In addition to making use of expression data, one recently developed strategy combines information from various sources to narrow down the search space in the network. This strategy shortens the time and effort required for the validation and discovery of networks. For example, if some gene names are known then they can be mapped into knowledge bases (e.g., the Gene Ontology) to extract biological knowledge (e.g., gene function) that can be used to determine the network structures. Our goal in this study is to establish a methodology for network inference and to investigate aspects of network dynamics that have not been addressed previously.

To infer a network with desired system behavior, the most important steps are to select a network model and then to fit the network's structural parameters to the available expression data. Many models have been proposed to address different levels of biological details, ranging from the very abstract (involving Boolean values only) to the very concrete (including full biochemical interactions with stochastic kinetics). Abstract models are easy to simulate and therefore less computationally taxing, but it has been proven that they are not able to capture certain system behaviors. In contrast, concrete models are more suitable for simulating biochemical process realistically, but, because of their computational complexities, these models can only be applied to small systems. To capture the underlying physical phenomena of a gene network we took one of the most popular and well-studied concrete models, the S-system model, as an example of a biological network, and we used both simulated and collected real gene expression data to reconstruct the model. The S-system model is a type of ordinary differential equation (ODE) model. It consists of a particular set of tightly coupled ODEs in which the component processes are characterized by power law functions [3,4]. The system structure of an S-system is described by the following equation:

$$\frac{dx_{i}}{dt}=\alpha_{i}\overset{N}{\underset{j=1}{\prod }}x_{j}^{g_{i,j}}-\beta_{i}\overset{N}{\underset{j=1}{\prod }}x_{j}^{h_{i,j}}$$

Here, *x_i _*is the expression level of gene *i*, and *N *is the number of genes in a genetic network. The non-negative parameters *α_i _*and *β_i _*are rate constants that indicate the direction of mass flow. The real-number exponents *g_i,j _*and *h_i,j _*are kinetic orders that reflect the strength of interactions from genes *j *to *i*. The above set of parameters defines an S-system model. To infer an S-system model is, thus, to estimate all of the 2*N *(*N*+1) parameters simultaneously.

Although non-linear ODEs can more accurately model the system dynamics of gene networks, they are difficult to solve by traditional optimization techniques [2,3]. To reduce the complexity of such models, Di Bernardo *et. al *developed an approach that involves taking a series of steady-state gene expression measurements following transcriptional perturbations and reducing the linear ODEs to a linear regression problem that can be solved with relevant techniques [5]. In addition, some researchers employed the strategies of gene clustering for dimension reduction and gene classification for identification of expression patterns to reduce task complexity [6-10]. As evolutionary algorithms (EAs) have been widely used to solve many difficult optimization tasks with good results, they have been suggested as a way to infer gene networks [3,4]. Among many studies on this topic, the most relevant are those involving the use of EAs to infer S-system models (e.g, [3,4,11]). In these studies, the network parameters of the S-system were arranged as a string of floating numbers (i.e., the chromosome in EAs) that could be evolved by genetic operators.

In recent studies of EA-based parameter inference, one critical problem, network robustness [12-14], has not been addressed. It is important to investigate the effect of network parameter perturbations on the overall system. These parameters not only are the numerical components of the model, but also represent the activities or interactions among proteins, transcription factors, and mRNAs in the gene network. Each network parameter has an important role in determining the behavior of a biological system. Parameters that are very sensitive to variation can introduce fragility into the system. In fact, recent research has found that parameters can define the dynamics of a model, and more important, studies have shown that enforcing constraints on the parameters can limit (control) model dynamics [15]. Specifically, by measuring and analyzing the variations of network parameters and their effects on relevant genes, the gene-gene interactions of an inferred model can be interpreted. To ensure the robustness of the inferred network and to further investigate gene interactions, it is important to derive an acceptable value range for each parameter and to restrict the parameter's value to the specified range during the network reconstruction process [13]. Therefore, we take parameter sensitivity into consideration in the network reconstruction procedure so that robust results can be obtained.

Sensitivity analysis (SA) is an indispensable technique that can help researchers to investigate the parameter robustness properties of an inferred network. By varying the parameter values within a certain range and performing statistical calculations to measure the system instability (or fragility), researchers can identify the critical parameters or discover seemingly unimportant parameters that may have a positive or negative influence on a network. SA techniques have been used in many biological studies, such as genetic circuit design [16], mammalian circadian clock modeling [17], and target prediction in signaling pathways [18]. In general, the sensitivity of a parameter is defined as [19]:

$$S_{P}^{M}=\frac{\partial M/M}{\partial P/P}=\frac{\text{percentage change in }M}{\text{percentage change in }P}$$

Here, *P *represents the parameters that are varied in a given range, *M *is the mathematical function describing the system behavior, and ∂*M* means the change in *M *due to the value changed in ∂*P* with respect to *P*.

In a dynamic system, there are two types of methods often used to conduct SA: local SA and global SA. Local SA can measure the sensitivity of a parameter from a given range even if the system structure is unknown, but it only considers one parameter at a time and ignores the interactions between parameters [20,21]. Alternatively, global SA can examine parameter interactions with different parameter magnitudes simultaneously [22,23], but this can only be done if the system structure is known in advance. The above two methods have been compared extensively [24,25]. It should be noted that neither local SA nor global SA is superior: the choice of method depends on the specific application and the information available. In the biological cases that involve the use of SA techniques, the sensitivity of parameters should be investigated from a biological point of view in order to validate the results.

From the perspective of parameter dynamics, we propose a new approach to infer robust networks. Our approach includes two major parts. The first part is a sensitivity analysis procedure that selects sensitive network parameters, determines value ranges for them, and then sends the parameters with their constraints to the inference mechanism. The second part is an evolutionary method responsible for inferring networks from the results obtained in the first part. This approach, to our knowledge, is the first work in network modeling that integrates sensitivity analysis into an inference algorithm to consider both internal (robustness) and external (behavior) characteristics of the inferred network. To validate the proposed approach, a series of experiments has been conducted, in which the proposed SA procedure was coupled with different EAs. The results have been analyzed, and they show that this approach can be used to infer robust networks successfully from artificial and real gene expression profiles.

## Methods

### Parameter sensitivity analysis

In the process of inferring a gene network from expression data the genes interact with each other, and the network structure is generally unclear during the modeling process. Most studies of global SA use known pathways as examples and can therefore easily select the critical parameters and control the total number of parameters (usually few than 100) for further analysis [23-25]. In the type of network inference problem addressed here, it is not possible to identify and select the most important parameters in order to perform global SA, because no prior information about network structure is available.

To consider multiple network parameters simultaneously in such situations, we present a new method that is a modification of a widely used global SA technique, called multi-parameter sensitivity analysis (MPSA) [12,19]. MPSA is based on Monte Carlo simulations and on quantitative comparisons of cumulative frequency via corresponding Pearson correlation coefficients among parameters to identify those that are most sensitive. It can be used to explore critical genetic reactions and to examine the robustness of a gene network as other global SA methods do. In the method described here, the objective function of the SA method is the fitness function that is to be optimized in the evolutionary algorithm, which is the mean squared error over the time course:

$$\overset{N}{\underset{i=1}{\sum }}\overset{T}{\underset{t=1}{\sum }}{\left\{\frac{x_{i}^{a}\left(t\right)-x_{i}^{d}\left(t\right)}{x_{i}^{d}\left(t\right)}\right\}}^{2}$$

In this expression, *x_i_^d^*(*t*) is the desired expression level of gene *i *at time *t*, *x_i_^a^*(*t*) is the actual value obtained from the inferred model, *N *is the number of genes in the network, and *T *is the number of time points used to measure gene expression data during the period.

Our modified method, *m*-MPSA, inherits qualities from MPSA but has several advantages. First, MPSA is easy to implement and modify. It allows users to select one parameter at a time or to consider multiple parameters at the same time to perform sensitivity analysis. This characteristic is crucial because, when the network structure is unknown or uncertain, selecting one parameter at a time is a good way to reduce the complexity of the search space. Researchers can then concentrate on each parameter and temporarily neglect the combination sets at the selection phase. Second, compared with most of the global SA methods, the mathematical equations of MPSA are more manifest and succinct, and the parameter sensitivity is thus easy to calculate. Finally, the value ranges of parameters used in MPSA are the same as in an ODE model. This means that the sensitivity of each parameter can be directly mapped into an ODE model without any extra computation.

In previous studies, researchers have shown that the most influential (sensitive) parameters play key roles in modeling a GRN; that is, a model's behavior varies largely because of its sensitive parameters [13]. Therefore, our current study draws on the characteristics of sensitive parameters, and it incorporates the sensitivity analysis method into the incremental evolution approach to infer a GRN. The proposed *m*-MPSA method is described in the ***Sensitivity analysis algorithm ***below. It uses an iterative process to calculate the sensitivity of each parameter, and it then ranks the sensitivities of all parameters. The input of this algorithm is the set of network parameters to be determined; and the output, a list of parameters ranked by their sensitivity values. With the parameter sensitivities ranked, the evolutionary algorithm can then infer robust solutions by exploiting the sensitive parameters that are likely to significantly influence the genetic model as a whole.

### Sensitivity analysis algorithm()

Step 1: Select one parameter, *i*, at a time. (*i *= 1, 2, .., *μ*, *μ *is the number of network parameters).

Step 2: Set the parameter range *R_i _*for each S-system parameter, *i*. Initially, the commonly used search regions [-3.0..3.0] and [0.0..10.0] are taken as the initial settings for the parameters of kinetic orders and rate constants respectively, as suggested in [26]. These settings are used to define the parameter range *R_i_*, by adding/subtracting an amount of one third of the initial settings to/from the current value of parameter *i*.

Step 3: For each *R_i_*, a set of random values are created with a uniform distribution within the specified range. Each random value for parameter *i*, together with the other (*μ*-1) parameters, constitutes a candidate solution (In these experiments, 500 random values were generated, as suggested in [19].

Step 4: Construct the mathematical model (i.e., S-system model) and calculate the objective function value for each of the random values generated in Step 3.

Step 5: Determine whether the objective function value of each random point obtained in Step 4 is acceptable or unacceptable by a given threshold *C_r _*(guided by the literature or experience; it is defined as the triple of the best available objective function value in the whole population). The parameter value of a random point is classified as *unacceptable *if its corresponding objective function value is greater than *C_r_*; otherwise, the parameter value is *acceptable*. Go back to Step 1 until all parameters have been dealt with.

Step 6: Calculate the sensitivity for parameter *i *by using its cumulative frequency (*CF_i_*). *CF_i _*measures the similarity of two statistical distributions that are formed by the acceptable and unacceptable values produced from Steps 3-5 for parameter *i*. The parameters are ranked according to their *CF*, and the ones with relatively low *CF *are considered to be sensitive.

Step 7: Output a parameter list in which the parameters are sorted by their CF values.

**Figure 2:**
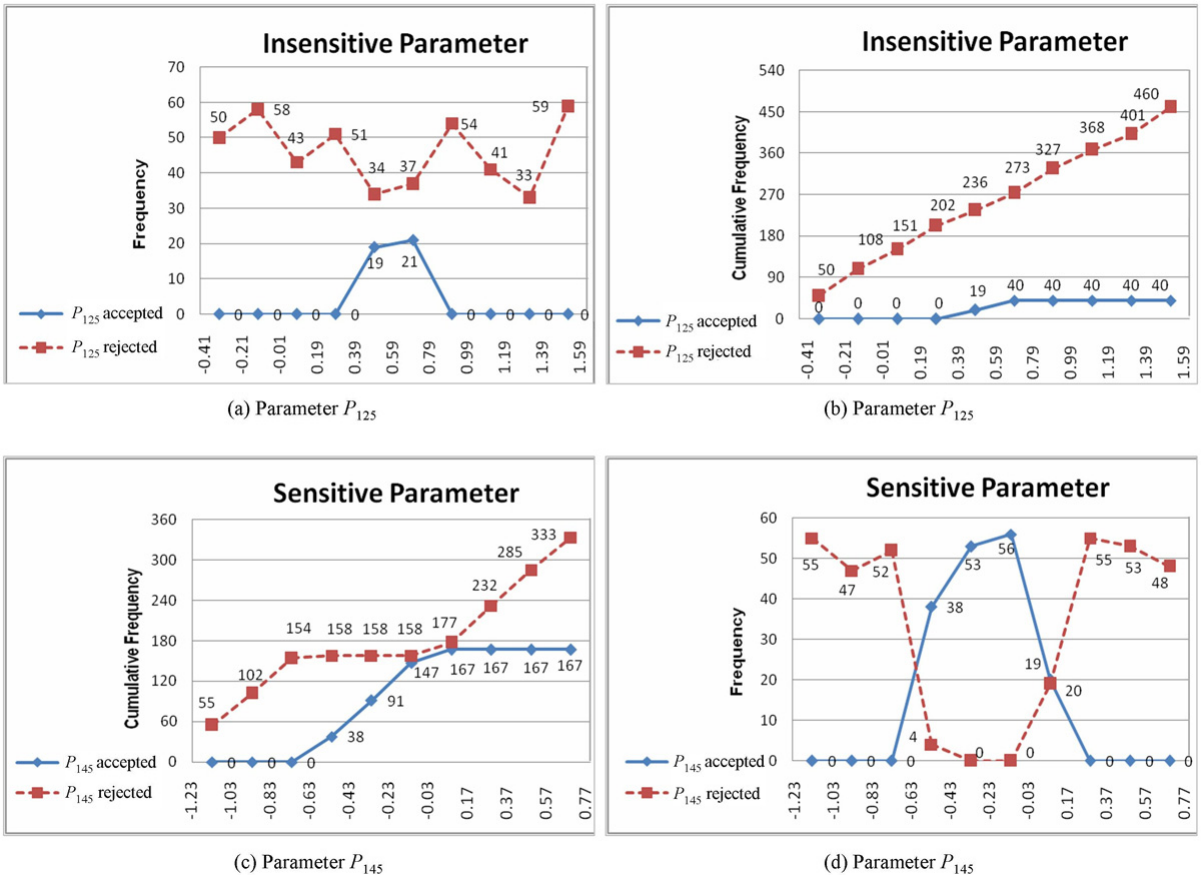
**An example of sensitivity analysis**. Example of using *m*-MPSA to obtain a sensitive parameter (*P*_145_) and an insensitive parameter (*P*_125_). The solid and dashed curves in both cases represent the acceptable and unacceptable results, respectively.

In addition to presenting the *m*-MPSA algorithm, we provide here a walkthrough example to illustrate how it operates in practice. In the example, we randomly choose two parameters from one of the datasets (the largest one) used in the experimental section, and we calculate the sensitivity values of these parameters. The dataset consisted of ten nodes, and there were altogether 220 parameters to be estimated. Without losing generality, we assume that the algorithm selected the parameter *P*_125 _(Step 1), and then set the range *R*_125 _for *P*_125 _(Step 2). As mentioned above, we used one third of the original search region (which was [-3.0, 3.0] in the current study) to set up the bounds for each parameter. Hence, if *P*_125 _had a value of 0.59, then the lower bound for this parameter was -0.41 (i.e., 0.59+1/3×(3.0-(-3.0))), and the upper bound was 1.59. Then, 500 random values were generated within the range [-0.41, 1.59] for *P*_125 _(Step 3), and their corresponding objective (fitness) function values were calculated (Step 4). Next, a threshold *C_r _*was determined to categorize the above 500 evaluation results as acceptable or unacceptable. If the current best result (out of the 500 evaluations) was 0.52, then a threshold of 1.56 (i.e., 0.52×3) was used to determine whether a result was acceptable or not (Step 5). After that, two statistical distributions were constructed for the acceptable and unacceptable results identified above. For statistical purposes, the value range [-0.41, 1.59] was divided into ten intervals, and for each interval the numbers of acceptable and unacceptable values were counted. Figure 2 (a) shows the distributions for *P*_125_, in which acceptable and unacceptable numbers were marked. The cumulative frequency distributions were then constructed accordingly, as presented in Figure 2 (b). Finally, the sensitivity value of *P*_125 _was obtained by measuring the correlation coefficients of the two (acceptable and unacceptable) cumulative frequency distributions (Step 6). In this case, the sensitivity value of *P*_125 _was 0.8912.

A similar procedure was performed for the other parameter, *P*_145_, and a sensitivity value of 0.7695 was obtained. Because *P*_145 _had a sensitivity value lower than that of *P*_125_, it was considered to be more sensitive than *P*_125_. In this way, after sensitivity values of all parameters were calculated, they were ranked and sent to the incremental evolution algorithm.

As mentioned above, more sensitive parameters are more likely to have a significant influence on the entire genetic model. Therefore, if no prior knowledge can be applied to the reconstruction of a large GRN, researchers can conduct sensitivity analysis for parameter selection to guide the network inference method, and observe how numerical quantities within different intervals can formulate candidate results (to determine the model dynamics). Taking *P*_145 _as an example, the acceptable values for this parameter fell in the range [-0.63, 0.17] that consisted of 167 acceptable network models (dynamics) in total. We can exploit this result to infer *P*_145 _and explore the relationships of this parameter with other genetic reactions (or parameters). In fact, researchers have shown that the most influential (sensitive) parameters play important roles in modeling a GRN. That is, the behavior variation of a network model largely depends on its sensitive parameters [13].

In Figure 2, we can see that the variation of the parameter value range influenced the system dynamics. That is, the system robustness was significantly affected by the value range specified for a parameter. For instance, the two distributions for *P*_145 _in Figure 2 (c) show that, if the value range of *P*_145 _changed from [-0.43, -0.23] to [0.37, 0.57], then the acceptable values for *P*_145 _changed from 56 to 0. This means that the inference algorithm can maintain the system robustness if it can locate *P*_145 _within the interval [-0.43, -0.23]. The above analysis concludes that the proposed SA method can be used to find sensitive parameters and to derive suitable value ranges as search constraints, which can then be used in the inference algorithm to construct robust networks.

### Evolutionary algorithm for parameter optimization

To derive the network parameters of S-system model, we also implement an evolutionary algorithm (EA) for parameter optimization to work with the above SA method. EA is a population-based approach that evaluates many solutions simultaneously in the search space, and it is likely to find a global solution for a given problem. Recently, a new population-based optimization technique, particle swarm optimization (PSO, [27]), was proposed as an alternative to the traditional EAs. PSO tries to mimic the goal-seeking behavior of biological swarms. The standard PSO algorithm contains a set of particles and operates in an iterative manner. Each particle is characterized by its position and velocity, and it moves in the solution space. The position of each particle represents a potential solution that is evaluated by a predefined fitness function. PSO has some attractive characteristics. In particular, it has memory, so that knowledge of a good solution can be retained by all particles (solutions). During the iterative search process, each particle remembers its previous best position and the best position of any particle in the swarm. Then the particle uses the position information to modify its position and velocity, and continues its movement in the search space. The details of the PSO algorithm can be found in [27]. Some performance comparisons between PSO and the most popular EA method, the genetic algorithm (GA), have been made, underscoring the reliability and the convergence speed of both methods. However, the result tends to be inconclusive: each of the algorithms has shown better performance than the other for some particular applications. Consequently, hybrid techniques were proposed to effectively exploit the qualities and the uniqueness of the two methods. It is now commonly agreed that the hybrid methods can lead to further performance improvements [28-30]. Therefore, in this work, we develop a hybrid method to exploit the solution memory of PSO and the genetic operations of GA for network inference.

As has been mentioned, our primary goal is to investigate how parameter sensitivity can be used to guide an EA and derive robust networks. To concentrate on this issue, we have chosen to implement a popular GA-PSO method (to avoid the deviations caused by different computational methods) for parameter optimization, and we have built our SA method upon it. Breeding Swarms [30] is a frequently used GA-PSO hybrid method that has demonstrated its good performance in many benchmark test functions. Therefore, we adopt this method for our network inference application.

**Figure 3:**
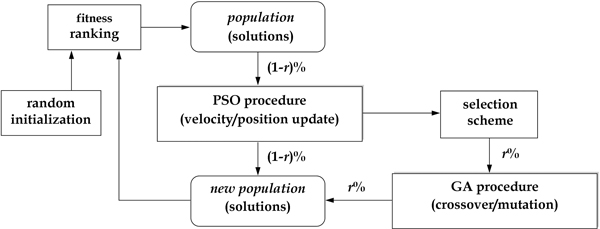
**GA-PSO method**. Flow diagram of the hybrid GA-PSO method used in this work.

The hybrid GA-PSO method used in this work is illustrated in Figure 3. Initially, a population is randomly generated and evaluated. The individual solutions are ranked according to their fitness values, and then they are divided into two parts for running the PSO and GA methods separately. As shown in Figure 3, the best individuals (including (1-*r*)% of the whole population) are preserved and enhanced by the PSO procedure. They are then sent to the next generation. Meanwhile, on the right-hand side of the figure, the remaining individuals (i.e., *r*% of the population) are discarded. To replace the removed individuals, a tournament selection scheme is used to choose the same number of individuals from the best ones (before they are updated by PSO), and the selected individuals are used to create new individuals by the GA procedure.

In our implementation, we take a direct encoding scheme to represent solutions for both the GA and PSO parts, in which the network parameters (i.e., *α_i_*, *β_i, _g_i,j_*, and *h_i,j _*in the S-system model) are arranged as a linear string chromosome of floating-point numbers. The goal here is to minimize the accumulated discrepancy between the gene expression data recorded in the dataset and the values produced by the inferred model. Therefore, the error (objective or fitness) function defined previously is used directly for performance measurement. For the PSO part, the equations for updating the particle's velocity and position are the same as the ones listed in the original PSO work [27], and for the GA part, the operations of crossover and mutation described in [31] are used.

### Sensitivity-based incremental evolution method for network inference

As described in the first section, to infer the S-system model is to determine the 2*N *(*N*+1) parameters simultaneously. Solving this high-dimensional problem is difficult, especially when the complexity of regulation increases along with the number of genes involved. One promising approach to this problem is to adopt the concept of incremental evolution to infer the large number of parameters. The underlying principle of incremental evolution [32,33] is that a population is first evolved to solve an easier version *T' *of the original complex task *T*, in which the solution region of *T *is more accessible from region *T'*. More task versions with incremental complexity can be arranged so that the original task can be achieved progressively. Evidently, the main task involved in implementing incremental evolution is the formulation of a scheme to transfer the goal task into another more evolvable task. In the process of task transformation, the structure of the environment and the overall goal must be preserved. This can usually be achieved by arranging the task sequence manually, or, alternatively, it can be done using an automated procedure. In this work we modify the cutting plane mechanism used in the high-dimension function optimization problem to develop an adaptive strategy to automatically perform incremental evolution.

In network inference, solving an easier version of the original task means evolving partial solutions (i.e., subsets of all network parameters) that can provide some guidance for the search and move toward the target solution gradually. In the proposed method, parameter sensitivities are calculated and used to determine the priorities of the parameters to be evolved (optimized). The sensitive parameters are selected iteratively, by the proposed SA method and the evolutionary method is employed to search the parameter dimensions accordingly.

**Figure 4:**
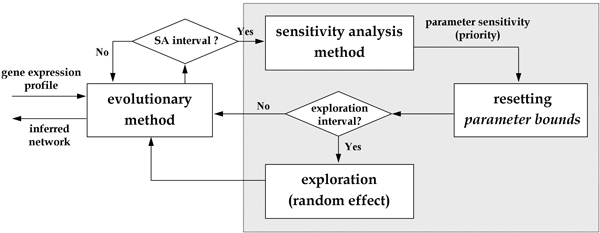
**The proposed approach**. Flow diagram of the proposed sensitivity-based incremental evolution approach.

An exploration phase is also included in the algorithm for the injection of random effects. This maintains population diversity and avoids a situation in which individuals move near locally optimal solutions. The SA and exploration procedures are both performed periodically with specific generation intervals. In this way, the overall solutions can be derived gradually, and the inferred networks are more robust to internal variations. The flow of the proposed SA-based incremental evolution approach is illustrated in Figure 4, and the details of the approach are described below.

### Sensitivity-based incremental evolution algorithm()

Step 1: Initialize the population and start the evolution. Note that Steps 2 and 3 are performed only at certain generations (SA interval and exploration interval, respectively).

Step 2: Perform sensitivity analysis (call the ***Sensitivity analysis algorithm ***described above) to obtain a list of parameters ranked by their sensitivity. This step is performed only if the evolutionary cycle reaches a pre-defined generation number (every 500 generations in this work).

(a) Use a threshold (i.e., the correlation coefficient ratio of the parameter CF values obtained from the SA procedure) to select the most sensitive parameters. Then update the threshold in order to consider more parameters in the next sensitivity analysis iteration.

(b) Set new value ranges for all network parameters. Sensitive parameters are given tight bounds, and insensitive parameters are given loose bounds.

Step 3: Start the exploration procedure, again only if the evolutionary cycle reaches a pre-defined generation number (every 1000 generations in this work). In this phase, a random value is generated for each parameter from its new bounds, replacing each parameter's current value.

Step 4: Continue the evolutionary computation, using the bounds obtained in Step 2 to restrict the corresponding parameters.

In the above algorithm, the most critical feature for enabling incremental evolution is found in Step 2. The strategy is to perform the sensitivity analysis for network parameters and to specify constraints on them. A network parameter (in other word, a search dimension) is added to the sensitive list and given a pair of tight constraints (specified by the upper and lower bounds) if its sensitivity value is less than (or equal to) the threshold; otherwise, a parameter is added to the insensitive list and given loose constraints. In the above procedure, the threshold used in Step 2(a) to identify the sensitive parameters has an initial value of 0.84 (chosen from the preliminary study), and this value is increased gradually with a step value of 0.1. The upper and lower bounds of a sensitive parameter in Step 2(b) are determined from the best value of this parameter available so far by adding and subtracting an amount equal to twice the value of a small constant (which is equal to the velocity bound generally used in a PSO-based method – 0.2 in this work). Bounds for the insensitive parameters are defined in a similar way, except that a value of five times the same constant is used.

The dimensions in the sensitive list have higher priorities to be searched, and the corresponding parameter values need to be determined at an earlier evolutionary stage. As the algorithm describes, the search is accomplished by setting small-range bounds for each sensitive parameter in accordance with the best value found so far, and the evolutionary procedure is performed next to infer a suitable parameter value within the specified bounds. The creation of constraints for parameters is important in the search for robust solutions, especially when a non-deterministic search method (such as the evolutionary algorithm) is employed to derive parameter solutions (because many feasible but fragile solutions could be evolved). If the parameter value exceeds the specified boundary value, the system dynamics will be influenced, and consequently, the network behaviors will change. To generate tight constraints for sensitive parameters in the search process is in fact to restrict search regions for the integrated algorithm, which can thus obtain robust networks with desired behaviors. As indicated in [34], by observing how parameters with varied boundaries direct a system to move toward different system dynamics, we can obtain new insights into a complex biological system and understand the principle of biological design.

As the evolutionary method proceeds, parameters in the insensitive list will be moved to the sensitive list incrementally as the threshold is gradually increased. Therefore, more parameters will be considered sensitive and their values will be determined. Eventually, all the parameter values will be obtained. Because sensitive parameters are more influential to network behavior, enforcing tight constraints on these parameters maintains the robustness of the entire genetic network. At the same time, it allows other parameters to evolve during the search process. In this way, the proposed method can infer robust network models with the desired system behavior.

## Results and discussion

To verify the proposed approach, we conducted a series of experiments on several datasets collected from the literature. As mentioned, our main goal was to explore the use of sensitivity analysis to infer robust networks, not to compare different computational methods. Therefore, without losing generality, we chose to implement three computational methods, including the traditional PSO method, a GA-PSO hybrid method, and a differential evolution (DE) method [35], and to build the proposed *m*-MPSA on them. The main reason to select the above three methods is that they are the most representative and popular evolutionary algorithms used for optimization tasks. The same SA strategy can also be embedded into other methods if their implementation details are available.

The first and second phases of the experiments involved comparing the external behavior and internal robustness of the networks inferred by different methods. Four datasets were used. For each dataset, the above three algorithms were performed with three different settings: (1) using only the original algorithms; (2) using our *m*-MPSA to select sensitive parameters and to derive parameter constraints in the incremental evolution process (i.e., the proposed approach); and (3) using *m*-MPSA to select sensitive parameters, but with random value bounds during the incremental evolution process. The third setting was mainly included to demonstrate the importance of using appropriate bounds in the evolutionary process. In addition, one real dataset was used in the third phase, which made it possible to investigate how the proposed approach can be applied to the study of real gene networks. This dataset came from a study of gene expression in the SOS DNA repair system of *E. coli*. For this dataset, network models were inferred and the critical parameters were analyzed and discussed.

### Performance of sensitivity analysis in network modeling

In this set of experiments, four datasets (with more genes than most examples encountered in the literature) were used to evaluate the proposed approach. The first dataset was a five-node artificial network that has become a popular model for comparing different methods in recent studies on network inference [36]. To collect time series data, we started network operations and continued operations for thirty simulation time steps. The nodes had the following relationships:

$$\begin{array}{c} {Ẋ}_{1}=15.0X_{3}X_{5}^{-0.1}-10.0X_{1}^{2.0}\\ {Ẋ}_{2}=10.0X_{1}^{2.0}-10.0X_{2}^{2.0}\\ {Ẋ}_{3}=10.0X_{2}^{-0.1}-10.0X_{2}^{-0.1}X_{3}^{2.0}\;\\ {Ẋ}_{4}=8.0X_{1}^{2.0}X_{5}^{-0.1}-10.0X_{4}^{2.0}\\ {Ẋ}_{5}=10.0X_{4}^{2.0}-10.0X_{5}^{2.0} \end{array}$$

The second dataset was taken from [6]. It was an eight-gene network created manually by the popular GRN simulation software tool Genexp. The third dataset was a ten-node network previously described in [4], given by the following equations:

$$\begin{array}{c} {Ẋ}_{1}=5.0X_{4}X_{6}^{-2.0}-10.0X_{1}^{2.0}\\ {Ẋ}_{2}=10.0X_{3}X_{8}^{1.0}-10.0X_{2}^{2.0}\\ {Ẋ}_{3}=8.0X_{1}^{-1.0}X_{4}^{-1.0}-10.0X_{3}^{2.0}\\ {Ẋ}_{4}=10.0X_{5}^{2.0}X_{9}-10.0X_{4}^{2.0}\\ {Ẋ}_{5}=10.0X_{2}^{2.0}X_{6}^{-1.0}-10.0X_{5}^{2.0}\\ {Ẋ}_{6}=5.0X_{9}^{2.0}X_{10}^{-2.0}-10.0X_{6}^{2.0}\;\\ {Ẋ}_{7}=10.0X_{6}X_{10}^{-1.0}-10.0X_{7}^{2.0}\\ {Ẋ}_{8}=5.0X_{1}X_{2}^{-2.0}X_{7}-10.0X_{8}^{2.0}\\ {Ẋ}_{9}=10.0X_{3}X_{8}^{-2.0}-10.0X_{9}^{2.0}\\ {Ẋ}_{10}=8.0X_{1}^{2.0}X_{7}^{-1.0}-10.0X_{10}^{2.0} \end{array}$$

The fourth dataset was part of a real experimental dataset from a study of the rat central nervous system (CNS) [37]. The original dataset included expression data for 112 genes collected at 9 time points (including the embryonic, postnatal, and adult stages). In this experiment, we selected a group of eight nodes representing the largest sub-network of the representative cluster reported in [6] as the target network. For the above four datasets, three inference algorithms (PSO, GA-PSO, and DE) with three different settings were arranged, and twenty independent runs were conducted for each arrangement. The population sizes for the four datasets were 800, 1000, 1600, and 1000, respectively. Tables 1, 2, 3, 4 show the results, in which the mean, standard deviation, and best and worst performance of the runs are listed for each arrangement.

**Table 1:** Fitness values for dataset 1

		PSO			GA-PSO			DE	
**Setting**	**1**	**2**	**3**	**1**	**2**	**3**	**1**	**2**	**3**

Avg	0.2845	0.0839	0.2502	0.0170	0.0054	0.0144	0.3150	0.1440	0.2856
Best	0.1801	0.0451	0.0997	0.0078	0.0020	0.0079	0.2485	0.1079	0.2452
Worst	0.4497	0.1442	0.3880	0.0236	0.0088	0.0215	0.3733	0.1820	0.3723
SD	0.1059	0.0327	0.0819	0.0048	0.0023	0.0053	0.0452	0.0237	0.0420

Fitness results obtained by three inference algorithms with different settings for dataset 1.

**Table 2:** Fitness values for dataset 2

		PSO			GA-PSO			DE	
**Setting**	**1**	**2**	**3**	**1**	**2**	**3**	**1**	**2**	**3**

Avg	0.5718	0.3647	1.5170	0.0589	0.0192	0.0769	2.7558	0.6857	1.8766
Best	0.3172	0.1315	1.1044	0.0310	0.0098	0.0530	2.2035	0.5169	0.9808
Worst	0.8492	0.4928	1.9613	0.1034	0.0314	0.1040	3.0571	0.9289	2.6480
SD	0.1729	0.1286	0.2743	0.0229	0.0062	0.0174	0.3933	0.1159	0.7201

Fitness results obtained by three inference algorithms with different settings for dataset 2.

**Table 3:** Fitness values for dataset 3

		PSO			GA-PSO			DE	
**Setting**	**1**	**2**	**3**	**1**	**2**	**3**	**1**	**2**	**3**

Avg	1.8992	1.0233	1.9141	0.3586	0.1404	0.2973	3.6997	1.8465	3.6311
Best	0.8746	0.7241	1.3902	0.2325	0.0799	0.2162	2.4568	1.6471	2.2708
Worst	3.7303	1.3419	2.2926	0.5930	0.1941	0.3671	4.7652	2.0209	4.3125
SD	1.0149	0.1925	0.2829	0.1035	0.0378	0.0465	0.7628	0.1328	0.6616

Fitness results obtained by three inference algorithms with different settings for dataset 3.

**Table 4:** Fitness values for dataset 4

		PSO			GA-PSO			DE	
**Setting**	**1**	**2**	**3**	**1**	**2**	**3**	**1**	**2**	**3**

Avg	0.6657	0.4732	1.1542	0.1661	0.1128	0.2280	1.7835	0.7052	1.8918
Best	0.4486	0.3224	0.8873	0.1373	0.0691	0.1320	1.1684	0.5572	1.3348
Worst	0.7960	0.5952	1.3679	0.2020	0.1376	0.3642	2.2143	0.8420	2.5387
SD	0.1341	0.0915	0.1487	0.0225	0.0199	0.0813	0.3024	0.1073	0.3962

Fitness results obtained by three inference algorithms with different settings for dataset 4.

As shown in Tables 1, 2, 3, 4, SA (setting 2) consistently outperformed the other two settings for inferring gene networks. It produced the best results for the average, best, and worst fitness values when used in conjunction with any of the three inference methods. Compared with other methods, the GA-PSO method produces smaller standard deviations, which indicates that it is more stable than the other methods. It should also be noted that the performance when using a *m*-MPSA method with random bounds (setting 3) was not as good as when using *m*-MPSA to select parameters and derive bounds for them. These results confirm the effectiveness of the proposed bound-setting strategy.

### Evaluation of network robustness

To evaluate the robustness of the networks inferred by three different settings for each algorithm, we compared fitness and the sensitivity values (averaged over all parameters for each network model) of the best solutions recorded from the final generations in all runs. Tables 5, 6, 7 list the results of using different settings for the three algorithms. The values recorded in the tables are the averaged results of the twenty runs performed for each setting. We can see that results for the four datasets presented in Tables 5, 6, 7 are consistent for all three inference algorithms. They confirm that the proposed SA-based approach (setting 2) is able to infer networks with lower fitness/error values (better system behavior) and lower sensitivity values (more robust S-system models) simultaneously. It should be noted that, because the three algorithms have different computational features (e.g., convergence rate, search strategy, and selection approach), the thresholds (relative values determined from the candidate solutions of each run) used for constructing the sensitive parameter list in each algorithm were not the same during the evolutionary process. Therefore, the sensitivities obtained from different algorithms are not directly comparable.

**Table 5:** Sensitivity values by PSO

	Setting 1		Setting 2		Setting 3	
	***Fitness***	***Sensitivity***	***Fitness***	***Sensitivity***	***Fitness***	***Sensitivity***

Dataset 1	0.2845	0.7622	0.0839	0.7203	0.2502	0.7554
Dataset 2	0.5718	0.7334	0.3647	0.7002	1.5170	0.7309
Dataset 3	1.8992	0.6865	1.0233	0.6463	1.9141	0.6620
Dataset 4	0.6657	0.7330	0.4732	0.7072	1.1542	0.7205

Comparisons of fitness and sensitivity values of the best solutions collected from the final generations. Experiments were conducted using the PSO method.

**Table 6:** Sensitivity values by GA-PSO

	Setting 1		Setting 2		Setting 3	
	***Fitness***	***Sensitivity***	***Fitness***	***Sensitivity***	***Fitness***	***Sensitivity***

Dataset 1	0.0117	0.7885	0.0054	0.7363	0.0144	0.7678
Dataset 2	0.0598	0.8249	0.0192	0.7581	0.0769	0.7867
Dataset 3	0.3586	0.8432	0.1404	0.7502	0.2973	0.7856
Dataset 4	0.1661	0.8084	0.1128	0.7691	0.2280	0.7802

Comparisons of fitness and sensitivity values of the best solutions collected from the final generations. Experiments were conducted using the GA-PSO method.

**Table 7:** Sensitivity values by DE

	Setting 1		Setting 2		Setting 3	
	***Fitness***	***Sensitivity***	***Fitness***	***Sensitivity***	***fitness***	***Sensitivity***

Dataset 1	0.3150	0.7901	0.1440	0.7438	0.2856	0.7791
Dataset 2	2.7558	0.7722	0.6857	0.7149	1.8766	0.7654
Dataset 3	3.6997	0.7709	1.8465	0.7027	3.6311	0.7509
Dataset 4	1.7835	0.7701	0.7052	0.7273	1.8918	0.7609

Comparisons of fitness and sensitivity values of the best solutions collected from the final generations. Experiments were conducted using the DE method.

Following the comparisons of the inferred networks, we used an example to further illustrate the effectiveness and importance of using sensitivity analysis to derive appropriate constraints for network parameters in the inference process. Here, we chose the third dataset (which was the largest network considered in the experiments) and investigated the corresponding parameter correlations. In this example of eight-gene network, there were 144 parameters (*P*_1_~*P*_144_) to be determined, of which the three most sensitive parameters, *P*_14_, *P*_64_, and *P*_72 _(identified as sensitive in at least fifteen out of twenty runs), were analyzed. Because the GA-PSO algorithm can give the best performance (as shown in Tables 5, 6, 7), data were collected from the runs using this method without (setting 1) and with (setting 2) the use of the SA method to derive value ranges for network parameters.

**Figure 5:**
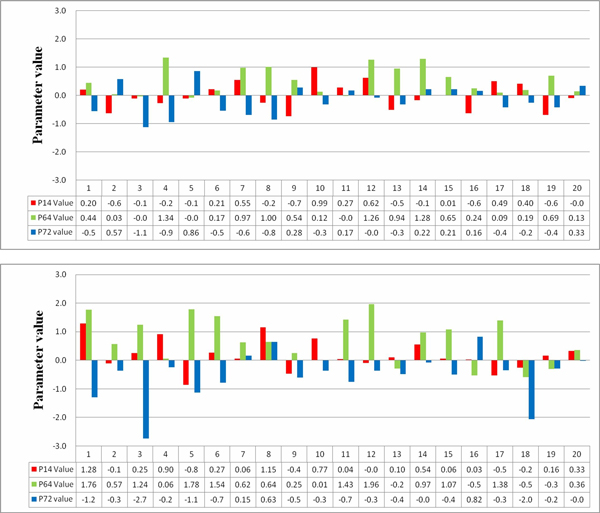
**An illustrated example**. The values of parameters *P*_14_, *P*_64_, and *P*_72 _obtained in the runs using setting 1 (upper part) and setting 2 (lower part).

The values of the above three parameters recorded from the runs are shown in Figure 5, in which the upper and lower parts are the results of the twenty runs conducted for settings 1 and 2, respectively. In this figure, the *x*-axis indicates the identity of a run, and the *y*-axis indicates the parameter values. In the results, we noticed two parameter correlations (or patterns) in the evolutionary process for each of the experimental settings. Table 8 lists the patterns most often obtained from the runs for the two settings (these patterns appeared in at least five runs). For setting 1, the two patterns (pattern-ns1 and pattern-ns2) describe two qualitative relationships: *P*_64 _>*P*_72 _>*P*_14 _and *P*_64 _>*P*_14 _>*P*_72_. For setting 2, the patterns are *P*_64 _>*P*_14 _>*P*_72 _(pattern-s1) and *P*_14 _>*P*_64 _>*P*_72 _(pattern-s2). The runs in which the patterns were observed are also indicated in the tables.

**Table 8:** Parameter patterns

Setting 1	run-id
pattern-ns1: *P*_64 _>*P*_72 _>*P*_14_	9, 13, 14, 15, 16, 19
pattern-ns2: *P*_64 _>*P*_14 _>*P*_72_	1, 4, 7, 8, 12

Setting 2	run-id

pattern-s1: *P*_64 _>*P*_14 _>*P*_72_	1, 2, 3, 5, 6, 9, 11, 12, 14, 15, 20
pattern-s2: *P*_14 _>*P*_64 _>*P*_72_	4, 8, 10, 13, 18

The parameter patterns obtained using two settings.

**Figure 6:**
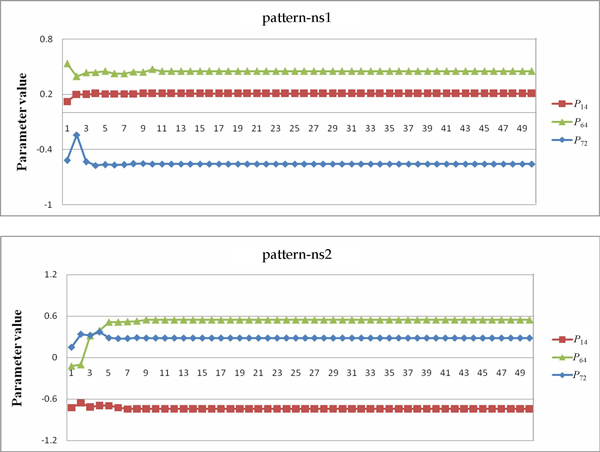
**GA-PSO with setting 1**. The variation of parameter values in a typical run of GA-PSO with setting 1.

**Figure 7:**
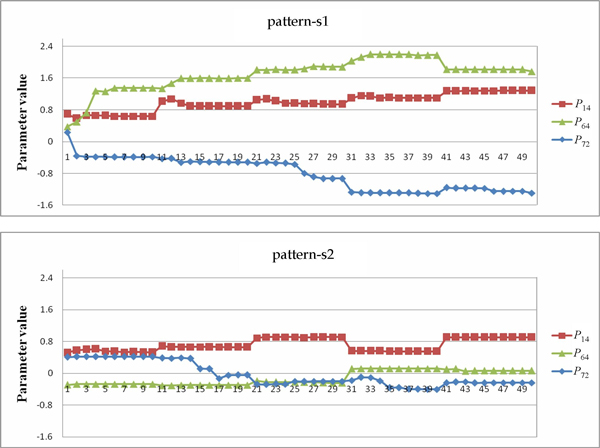
**GA-PSO with setting 2**. The variation of parameter values in a typical run of GA-PSO with setting 2.

In Table 8, we can see that the qualitative relationship *P*_64 _>*P*_14 _>*P*_72 _appeared in the results for both settings (pattern-ns2 and pattern-s1). After further investigation, we found that this relationship among the three parameters was important for producing good models (models with low fitness/error and high robustness). Using our specially designed SA method and parameter constraints, this relationship can always be derived and kept in the evolutionary process (in eleven out of twenty runs), but it is not often obtained in the runs with setting 1 (only five out of twenty runs with setting 1 showed the relationship). Similarly, another parameter relationship, *P*_14 _>*P*_64 _>*P*_72 _(pattern-s2), obtained from setting 2, was useful for inferring good models. To observe how the three parameters varied during the runs, see Figures 6, 7. These figures illustrate typical examples of the patterns presented in Table 8. In the figures, the *x*-axis indicates the generation number (each unit represents 100 generations), and the *y*-axis indicates the parameter value. As can be clearly seen in Figures 6, 7 the parameter values changed actively to move toward the appropriate positions to obtain a successful model in the runs using setting 2, whereas the values remained static in the runs using setting 1. These results verify the effectiveness of using SA in network inference.

### Evaluation of the proposed approach on a real dataset

After evaluating the performance of the proposed SA-based approach in network inference, we conducted a set of experiments to investigate how our approach can be applied to the study of a real gene network. Because the GA-PSO algorithm has been shown to outperform other methods in the above experiments, we used this method with two settings (with and without SA) to conduct experiments on a real dataset.

**Figure 8:**
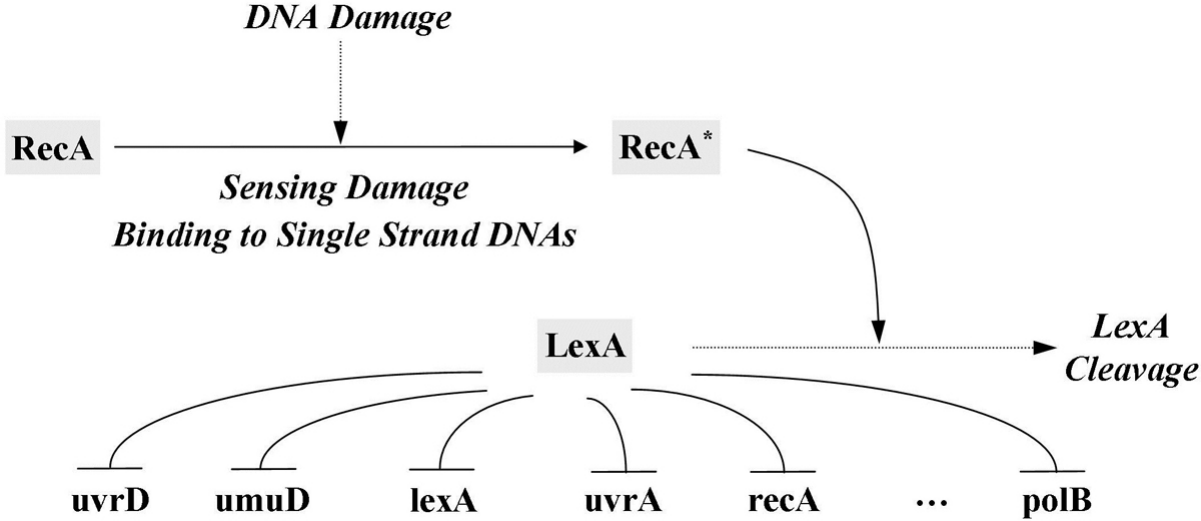
**SOS DNA repair system in *E.coli***. SOS DNA repair system in **E.coli **(-| means inhibition and → means activation). When DNA damage occurs, the RecA protein becomes activated and mediates cleavage of the LexA protein. The repair system is activated by a drop in LexA concentration.

The data set used in this experimental phase comes from a study of the SOS DNA repair system in *E. coli*. Figure 8 illustrates the gene regulation that occurs in this system [38]. In this system, the LexA protein (a repressor) maintains its expression level in a normal state and is bound to the promoter regions of SOS genes, including uvrD, umuD, lexA, uvrA, recA, and polB. When DNA damage occurs, the RecA protein senses the damage and mediates LexA autocleavage. The decrease in LexA relieves the repression of the SOS genes. The expression of these genes then activates the SOS repair system. Once the damage has been repaired, the concentration of recA drops and this gene stops mediating LexA autocleavage. The LexA level increases and begins to repress the SOS genes again.

#### Inference of expression profiles with the decomposed S-system model

In the experiments, six genes were selected from the original experimental data reported in [39]. The genes selected were uvrD, umuD, lexA, uvrA, recA, and polB. These were selected because the interactions of the 6-gene network have been well studied and commonly used in related studies, and the corresponding network has been inferred successfully [11,40-43]. Using the regulatory relationships that have been described, we can validate our proposed network inference method by comparing our results to those reported previously.

Though there have been several studies on inferring the SOS repair system, none of them used a tightly coupled S-system model (the general S-system) to represent the gene network, and none of them inferred such a model from expression data. To the best of our knowledge, the most relevant work is [44], in which the authors utilized a decoupled S-system model to infer the SOS repair system. In a decoupled S-system, a tightly coupled system of non-linear differential equations is decomposed and analyzed as several differential equations [44,45], each of which can describe a specific gene and can then be separately inferred (by considering one gene at a time). Motivated by this research, we thus adopted decoupled differentials to describe gene profiles, and we employed the proposed approach to infer gene interactions. In this way, we can not only examine the inferred network behaviors, but also compare the inferred gene regulations to the ones obtained in the other studies mentioned above.

**Figure 9:**
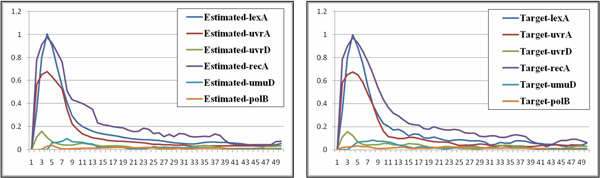
**Overview network behavior of the SOS repair system**. The inferred (left) and target (right) network behaviors of the SOS repair system (An overview).

**Figure 10:**
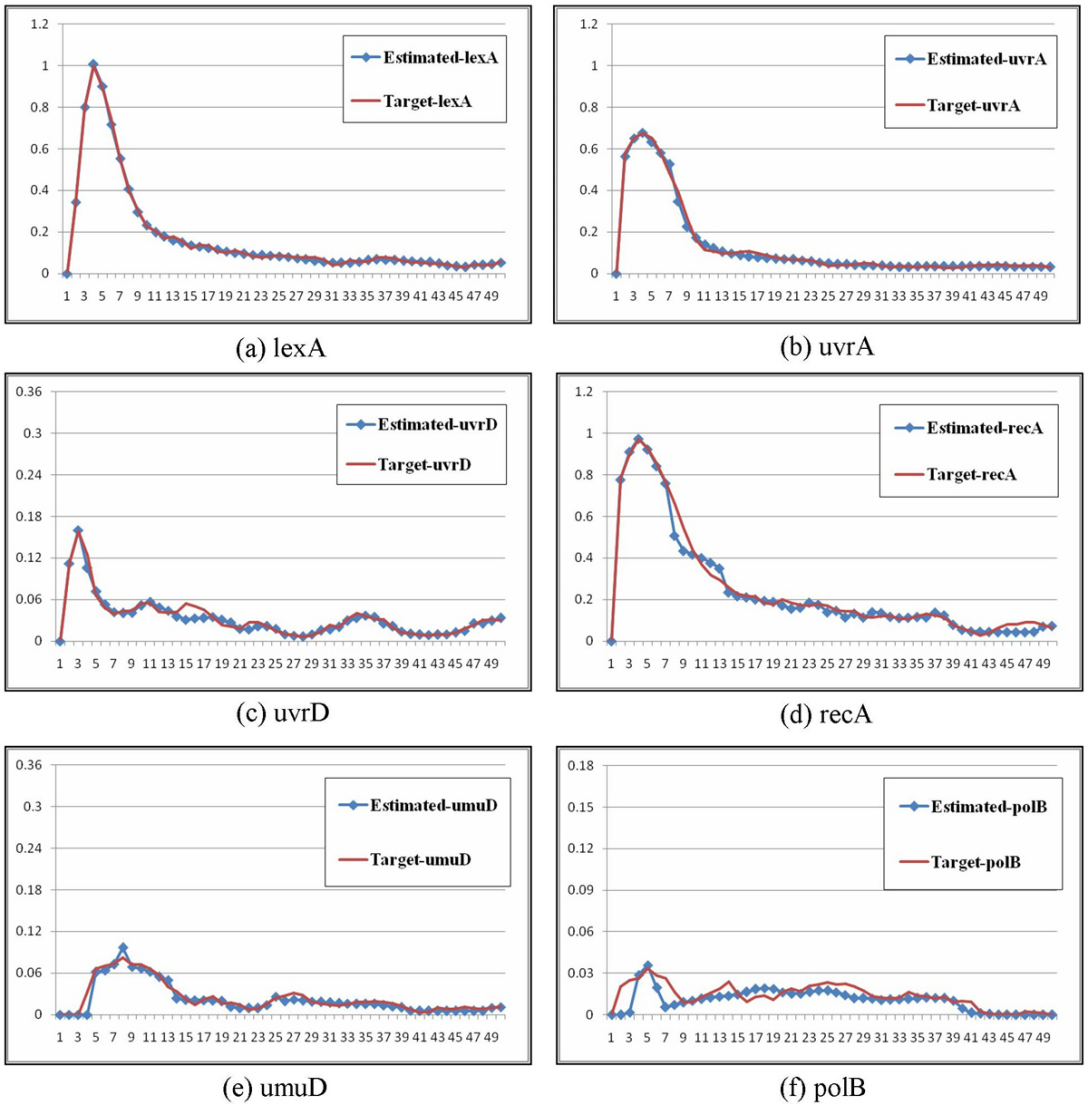
**Gene expression in the SOS repair system**. The target and inferred profiles of the six genes in the SOS repair system.

The SOS repair system dataset included 50 sampling points for each gene. The six major genes (lexA, uvrA, uvrD, recA, umuD, and polB) were inferred through the decoupled S-system model. In the experiments, twenty independent runs were conducted for each of the two settings. Table 9 presents the results for the six genes of the SOS repair system dataset obtained by using settings 1 and 2. The mean, variance, best and worst fitness, and average parameter sensitivity values of the twenty runs are listed. Again, the results show that the SA-based approach (setting 2) outperformed the original algorithm in terms of both fitting the external behavior (with lower fitness/error) and exploring the internal robustness (with lower sensitivity) of a gene network. In addition, to observe the inferred network behaviors, Figures 9, 10 compare the inferred and target behaviors. In these figures, the *x*-axis represents time, and the *y*-axis represents the concentrations of particular gene products. As shown in the figures, network behaviors very similar to those of the real system can be inferred by our approach.

**Table 9:** Results by GA-PSO

	lexA		uvrA		uvrD	
**Setting**	**1**	**2**	**1**	**2**	**1**	**2**

Avg	1.5778	0.5360	1.8404	0.7447	4.0382	1.3453
Best	0.6302	0.4084	1.1121	0.5333	1.6084	1.2191
Worst	1.9917	0.9554	2.1375	1.2190	7.4100	1.5640
SD	0.4145	0.1974	0.3572	0.2084	2.1226	0.1130
Sensitivity	0.8355	0.7977	0.8417	0.7838	0.7890	0.7890

	**recA**		**umuD**		**polB**	

**Setting**	**1**	**2**	**1**	**2**	**1**	**2**

Avg	3.0527	1.8589	6.0470	4.1333	21.1221	14.6093
Best	2.4037	0.9570	3.9336	3.9694	16.4665	10.7421
Worst	3.8859	2.2015	7.4803	4.5852	25.3341	16.6921
SD	0.5531	0.4623	1.0923	0.1756	2.7867	2.0940
Sensitivity	0.8669	0.7943	0.8315	0.7841	0.8507	0.7787

Results obtained by the GA-PSO algorithm with two settings.

#### Analysis of critical parameters of the SOS repair system

**Figure 11:**
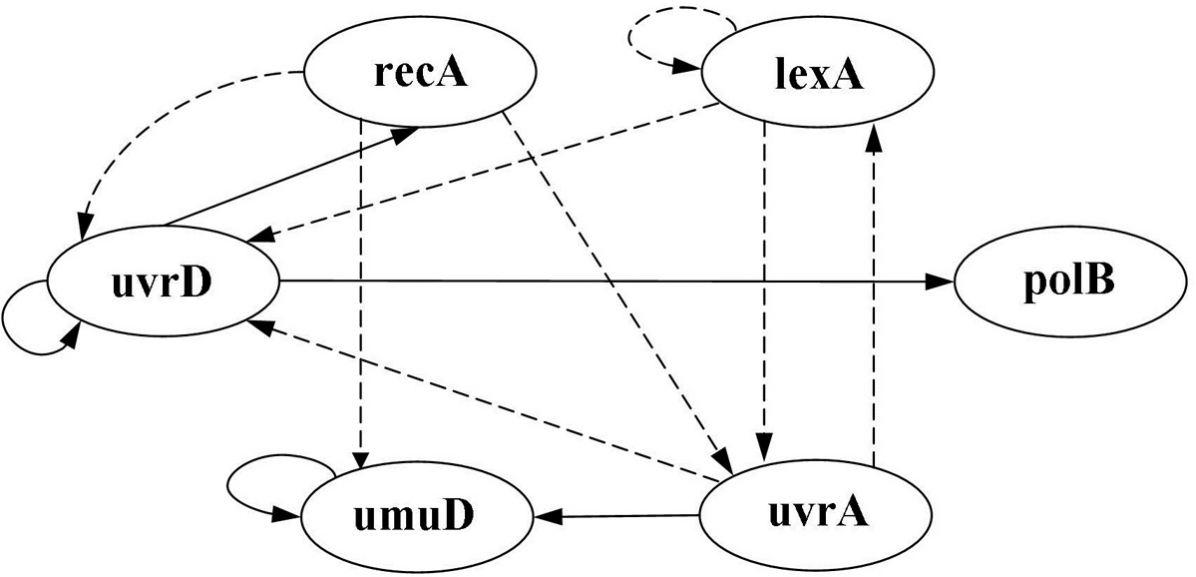
**Inferred network structure**. Structure of the 6-gene SOS repair system inferred by the proposed approach (dashed lines indicate inhibition; solid lines indicate activation).

To investigate the critical parameters that have significant influences on the system dynamics of the SOS repair system, we summarized the most sensitive parameters identified by the proposed approach in Table 10. There were 13 parameters selected and marked as crucial interactions, each of which represented a regulatory relationship between two genes (e.g., lexA -| uvrA). The letters G and H in the table indicate which of the kinetic orders *g_i,j _*and *h_i,j _*of gene *i *(as listed in the first column) is selected. As mentioned previously, the identified parameters (or gene regulations) can also be used to determine the network structure of the system to be inferred. By exploiting the structural information, the evolutionary algorithm (here, GA-PSO) can infer better models with robust system dynamics during the search process. In the SOS case, the 13 parameters indicated in Table 10 can be used to derive the scaffold of the SOS network, as shown in Figure 11.

**Table 10:** The most sensitive parameters

Gene-id (*i*)	Gene name	Num of related gene	*g_i_*_,1_/*h_i_*_,1_	*g_i_*_,2_/*h_i_*_,2_	*g_i_*_,3_/*h_i_*_,3_	*g_i_*_,4_/*h_i_*_,4_	*g_i_*_,5_/*h_i_*_,5_	*g_i_*_,6_/*h_i_*_,6_	*g_i_*_,7_/*h_i_*_,7_	*g_i_*_,8_*/h_i_*_,8_
			
			lexA	uvrA	uvrD	recA	uvrY	ruvA	umuD	polB
1	lexA	2	H*	H*						
2	uvrA	2	H*			H*				
3	uvrD	4	H*	H*	G	H				
4	recA	1			G*					
5	umuD	3		G*	G	H*				
6	polB	1			G*					

The most sensitive parameters identified by the proposed approach. The letter G indicates that *g_i,j _*is the most influential parameter for gene *i*; while the letter H indicates that *h_i,j _*is the most influential parameter. The asterisk means the regulation known in the literature (See Table 11 for details).

To compare the gene regulation events identified by our approach to those found in the literature, we highlighted the gene interactions in Table 10 (the asterisks in the table are the ones collected from the literature) and summarized the results in Table 11. The results show that, of the thirteen gene regulation events found in our experiments, ten matched regulatory relationships known from other studies. For example, in Figure 11, the negative regulation of lexA, uvrA, and uvrD by lexA has been successfully identified as the most crucial interaction in determining system dynamics. Still, it is notable that the known negative regulation of lexA by recA was not recognized. The main reason for this is that our approach chose sensitive parameters from the kinetic orders of the S-system model on the gene level, but the above relationship was in fact interpreted as a regulation of protein LexA by protein RecA [11,43]. The concentration of protein RecA depends on the sensing of DNA damage, and the interaction between recA and lexA therefore depends on the events occurring between DNA damage and DNA repair. Because the *on *or *off *state of DNA damage sensing was not directly described in the S-system model, the regulation of lexA by recA on the protein level was not observable on the basis of kinetic orders. In other words, the absence of this regulation was due to the model representation, rather than the inference approach. This shows that, to infer the SOS repair system accurately on both the protein and gene levels, a more comprehensive mathematical model is needed to describe the regulatory details. With only one exception (mentioned above) that was caused by limitations of the model itself, our approach has shown its strength in inferring real gene system that show the expected network behavior and internal network robustness.

**Table 11:** Critical gene regulatory relationships

Gene-id (*i*)	Gene name	Gene regulation	References
1	lexA	lexA -| lexA (*h*_1,1_), uvrA -| lexA (*h*_1,2_)	[11,40,43,44]
2	uvrA	lexA→uvrA (*g*_2,1_), recA→uvrA (*g*_2,4_)	[43,44]
3	uvrD	lexA -| uvrD (*h*_3,1_), uvrA -| uvrD (*h*_3,2_)	[11,41,43]
4	recA	uvrD→recA (*g*_4,3_)	[43]
5	umuD	uvrA→umuD (*g*_5,2_), recA -| umuD (*h*_5,4_)	[11,43]
6	polB	uvrD→polB (*g*_6,3_)	[44]

Summary of critical gene regulatory relationships obtained by the proposed framework with respect to the results in the literature. The parameters *g_i,j _*and *h_i,j _*are kinetic orders of the S-system.

After identifying the most sensitive parameters of the SOS repair system, we again analyzed the gene regulation relationships obtained from the runs to observe the correlation of parameters. The importance of investigating the relationships among parameter values of genes has been emphasized in the study of systems biology, especially when the boundary of a parameter value is taken into consideration [15,34]. As presented and discussed in the second experimental phase, the qualitative relationships among genes can be regarded as special patterns (describing parameter correlations) that determined the system dynamics of a candidate model. By exploiting these patterns, the inference algorithm can refine the values of kinetic orders to obtain better solutions.

**Figure 12:**
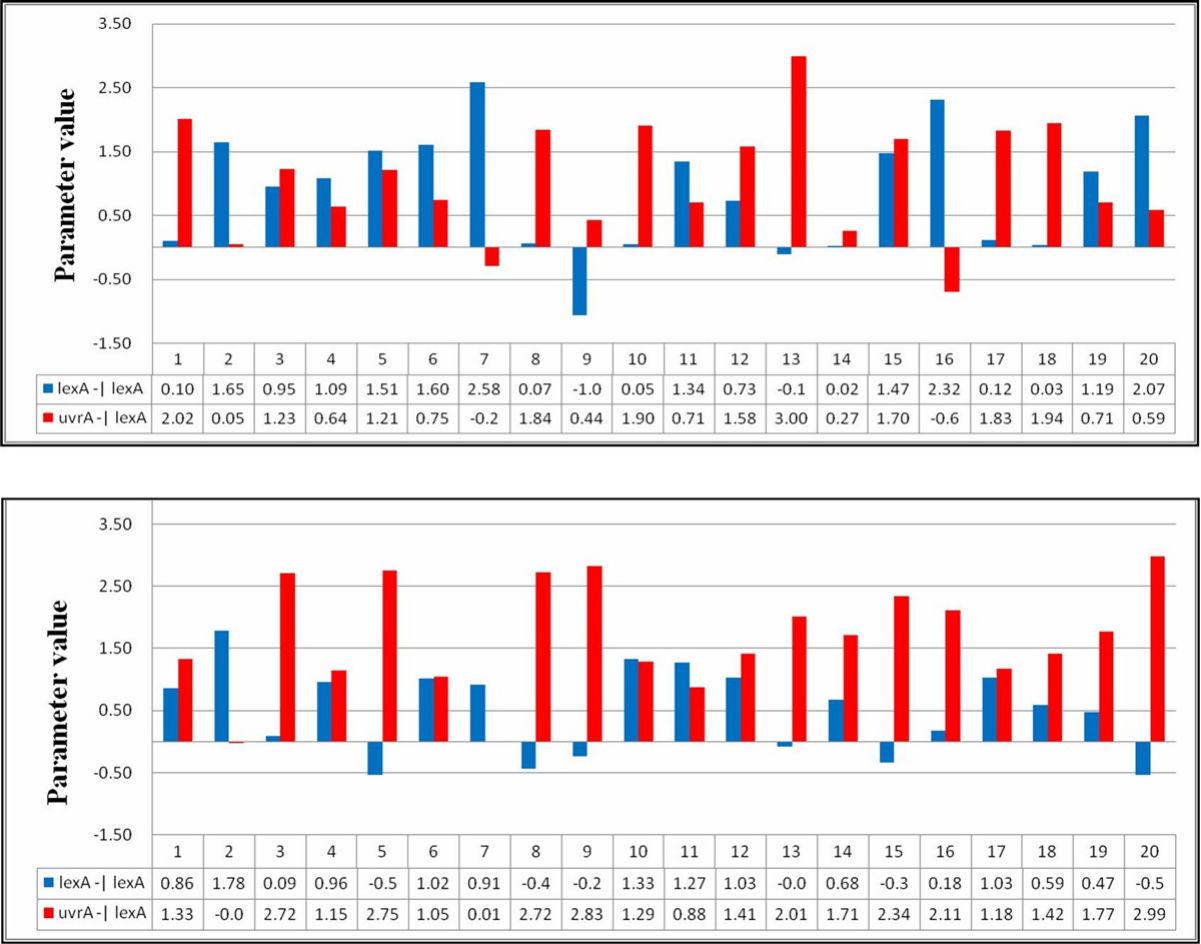
**Parameter values for lexA**. The parameter values related to the patterns found from the runs for lexA. The upper and lower parts are results for settings 1 and 2, respectively.

In the case of the SOS repair system, the patterns of regulatory interactions can be derived as in other cases, by recording and analyzing the values of the most sensitive parameters. Taking gene lexA as an example (see Table 12), the algorithm categorized genes lexA and uvrA as the critical gene regulators for lexA and two regulatory relationships, lexA -| lexA and uvrA -| lexA can be established on the basis of this information. Figure 12 illustrates the parameter values related to the patterns derived from the twenty runs. In this figure, the *x*-axis indicates the run number, and the *y*-axis represents the parameter values of the specific pattern (gene regulation) obtained in each run. In this figure, the upper and lower parts present the results of inferring the network without (setting 1) and with (setting 2) using the SA method, respectively. The regulation patterns for gene lexA are summarized in Table 12. As shown in Table 12, for experimental runs using setting 2, fifteen out of twenty runs consistently produced the same pattern uvrA -| lexA > lexA -| lexA (pattern-s1). This pattern can guide the inference algorithm to find better solutions. In contrast, the qualitative relationships between the two relationships uvrA -| lexA and lexA -| lexA obtained from the runs using setting 1 are inconsistent and unstable (pattern-ns1 and pattern-ns2 are in fact complementary). The above results show that the proposed approach can derive parameter bounds (which form useful patterns) to restrict the variation of lexA, and it can therefore infer models with better fitness and more stable (robust) system dynamics. Without using the SA method, the inference algorithm alone cannot guarantee the preservation of useful patterns during the iterative inference process.

**Table 12:** Gene regulations for lexA

Parameter patterns for gene lexA	
Setting 1	run-id

pattern-ns1: uvrA -| lexA > lexA -| lexA	1, 3, 8, 9, 10, 12, 13, 14, 15, 17, 18

pattern-ns2: lexA -| lexA > uvrA -| lexA	2, 4, 5, 6, 7, 11, 16, 19, 20

Setting 2	run-id

pattern-s1: uvrA -| lexA > lexA -| lexA	1, 3, 4, 5, 6, 8, 9, 12, 13, 14, 15, 16, 17, 18, 19

The patterns of gene regulations found for target gene lexA.

**Figure 13:**
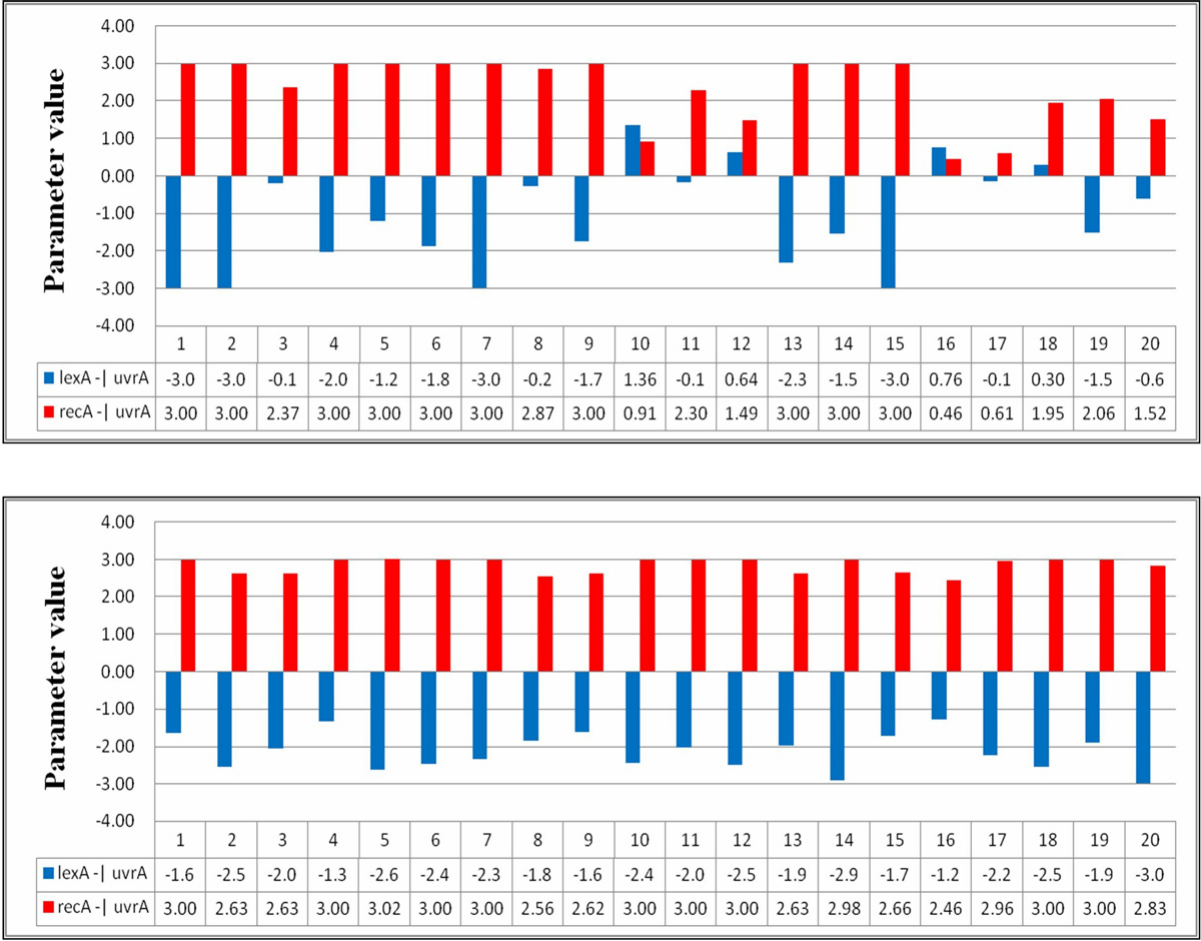
**Parameter values for uvrA**. The parameter values related to the patterns found from the runs for uvrA. The upper and lower parts are results for settings 1 and 2, respectively.

**Figure 14:**
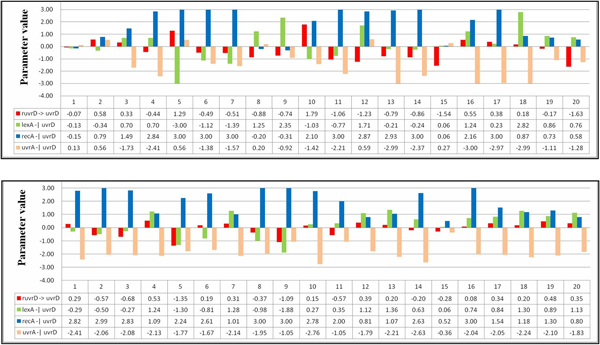
**Parameter values for uvrD**. The parameter values related to the patterns found from the runs for uvrD. The upper and lower parts are results for settings 1 and 2, respectively.

**Figure 15:**
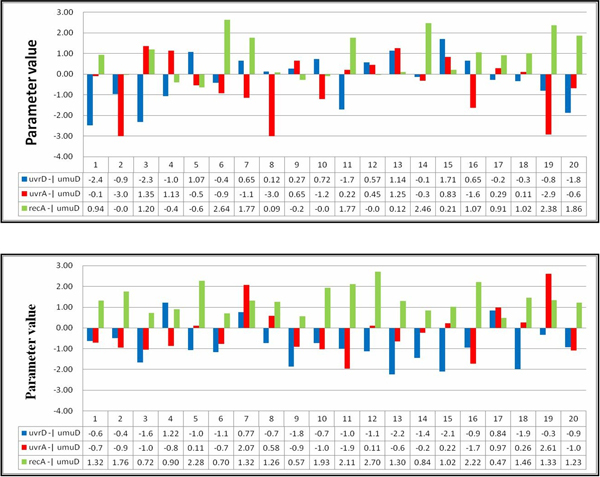
**Parameter values for umuD**. The parameter values related to the patterns found from the runs for umuD. The upper and lower parts are results for settings 1 and 2, respectively.

Tables 13, 14, 15 list the regulation patterns analyzed for four other target genes in the SOS system: lexA, uvrA, uvrD, and umuD. Figures 13, 14, 15 show the parameter values involved. The genes recA and polB were ignored because there was only one gene regulatory relationship found for each of them (uvrD→recA for recA, and uvrD→polB for polB, as shown in Table 11). This means that no qualitative relationship can be established for these genes. The results in Tables 13, 14, 15 are consistent with Table 14, which again shows the success of using SA in network inference.

**Table 13:** Gene regulations for uvrA

Parameter patterns for gene uvrA	
Setting 1	run-id

pattern-ns1: recA→uvrA > lexA→uvrA	all runs except for 10 and 17

Setting 2	run-id

pattern-s1: recA→uvrA > lexA→uvrA	all twenty runs

The patterns of gene regulations found for target gene uvrA.

**Table 14:** Gene regulations for uvrD

Parameter patterns for gene uvrD	
Setting 1	run-id

pattern-ns1: recA -| uvrD > lexA -| uvrD > uvrD-| uvrD > uvrA -| uvrD	3, 4, 11, 13, 14, 16

Setting 2	run-id

pattern-s1: recA -| uvrD > lexA -| uvrD > uvrD-| uvrD > uvrA -| uvrD	2, 3, 5, 10, 11, 14, 15, 16, 17, 19

pattern-s2: lexA -| uvrD > recA -| uvrD > uvrD-| uvrD > uvrA -| uvrD	4, 7, 12, 13, 18, 20

The patterns of gene regulations found for target gene uvrD.

**Table 15:** Gene regulations for umuD

Parameter patterns for gene umuD	
Setting 1	run-id

pattern-ns1: recA -| umuD > uvrD -| umuD > uvrA -| umuD	2, 6, 7, 14, 16, 19

pattern-ns2: recA -| umuD > uvrA -| umuD > uvrD -| umuD	1, 11, 17, 18, 20

Setting 2	run-id

pattern-s1: recA -| umuD > uvrA -| umuD > uvrD -| umuD	3, 5, 6, 8, 9, 12, 13, 14, 15, 18

pattern-s2: recA -| umuD > uvrD -| umuD > uvrA -| umuD	1, 2, 10, 11, 16, 20

The patterns of gene regulations found for target gene umuD.

## Conclusion

In this work, we developed a sensitivity-based incremental evolution approach to cope with one important issue, network robustness, which has not been addressed in gene network inference. Our approach included two parts. The first part was a sensitivity analysis method that was used to select sensitive parameters for deriving value bounds of these parameters. The second part was an evolutionary algorithm that took the bounds as constraints to perform parameter optimization. This process leads to inferred networks that are robust and produce the desired behaviors. To validate the proposed approach, a series of experiments were conducted to evaluate the external behaviors and internal robustness of the networks inferred by different methods. The results show that the proposed SA-based approach outperformed other methods in all datasets. Moreover, we analyzed in detail the results obtained from real time-series expression data for the SOS repair system. The analyses indicate that our approach can identify the critical parameters and use them to establish regulatory relationships among genes. By enforcing these relationships in the repetitive search process, our approach can successfully infer robust networks.

## Competing interests

The authors declare that they have no competing interests.

## Authors' contributions

YH undertook the experimental implementation, made statistical analysis and wrote a part of the manuscript. WL conceived the project, designed the algorithm, wrote a part of the manuscript, and made modifications as well as final revisions.
